# Neurological involvement, immune response, and biomarkers in Kawasaki disease along with its pathogenesis, therapeutic and diagnostic updates

**DOI:** 10.12688/f1000research.130169.2

**Published:** 2023-04-03

**Authors:** Omniat Amir, Priyadarshi Prajjwal, Pugazhendi Inban, Srikanth Gadam, Soumya Aleti, Rayyan Rafik Sunasra, Karan Gupta, Mustafa Elhag, Mohammed Mahmoud, Omklthoum Alsir

**Affiliations:** 1 Medicine, Almanhal academy, Khartoum, Sudan; 2 Neurology, Bharati Vidyapeeth University Medical College and Hospital, Pune, India; 3 Medicine, Government Medical College Omandurar, Chennai, India; 4 Postdoctoral Research Fellow, Mayo Clinic, USA, USA; 5 Internal Medicine, Berkshire Medical Center, Pittsfield, Massachusetts, USA; 6 Medicine, Hinduhriday Samrat Balasaheb Thackeray Medical College, Mumbai, India; 7 Orthopedics, Government medical college, Patiala, India

**Keywords:** Kawasaki, Strawberry Tongue, Rash, Inflammation

## Abstract

Kawasaki disease is an acute, febrile disease that is not typically fatal if treated and affects infants and children more commonly. More than 80% of the afflicted patients are under the age of four. This disease most commonly affects coronary arteries. In a minority of cases, Aneurysms can burst or produce thrombosis, and they can cause infarction. The distinctive redness in the palms and soles of the feet might result from a delayed-type hypersensitivity reaction to a cross-reactive or recently discovered antigen (s). Autoantibodies against epithelial cells and smooth muscle cells are produced as a result of subsequent macromolecule synthesis and polyclonal white blood cell activation, which intensifies the redness. Kawasaki disease's clinical manifestations range from oral skin disease to the blistering of the mucosa, symptoms involving the hands and the feet, skin disease of the palms and soles, a desquamative rash, and cervical lymphatic tissue enlargement (so it is also referred to as tissue layer lymphatic tissue syndrome). Most untreated patients develop some vessel sequelae, from well-organized coronary inflammation to severe arterial blood vessel dilatation to giant artery aneurysms with rupture or occlusion, infarction, and thrombosis. With human gamma globulin administration, reasonable standards of medical care, and the use of analgesics, the speed of symptomatic progression and inflammatory artery changes are reduced. In this review, we have covered the immunology of Kawasaki disease, its biomarkers, and the neurological manifestations of this multisystem illness. We have also included a discussion on its pathogenesis, diagnosis, and treatment.

## Introduction

Dr. Tomisaku Kawasaki originally recognized Kawasaki disease (KD) as a spontaneous lymphoid tissue illness in 1967. It is an abrupt, widespread inflammation with unclear causes that primarily affects newborns and young children. Japan has a high prevalence of KD, with 264.8/1,000,000 children under 5 receiving treatment there in 2012. A major essential KD consequence that affects exactly 25% of untreated individuals is arterial problems. ^1^ Combining KD designation patterns shows seasonality, with incidence peaks that vary based on demographic studies.

It should be noted that this sickness typically peaks in the winter and spring when illnesses caused by metabolic infectious agents coincide with these seasons. Compared to Japan, where the incidence rate of KD is 330 per 100,000 children aged 0–4 years annually, the United States reports a hospitalization rate of 20 per 100,000 children aged 0–4 years. White Americans and children of Asian origin living in Japan are most at risk, whereas Pacific and Asian heritage residing in the United States are impacted at a moderate incidence. Therefore, any genetic or ethnic origin might potentially impact the etiology of KD. ^2^ Kawasaki disease, being a multisystem illness, can have manifestations in various systems of the body including the central nervous system. The central nervous system manifestations include seizures, facial paralysis, hemiplegia, myositis, etc. It is important to ensure the correct diagnosis and commence proper treatment in order to prevent complications like cerebral infarction or aneurysm. The purpose of this review is to give an overview of the pathogenesis, immunology, and diagnostic biomarkers of KD and help healthcare individuals recognize it early to prevent any complications.

## Methods

**Figure 1.**
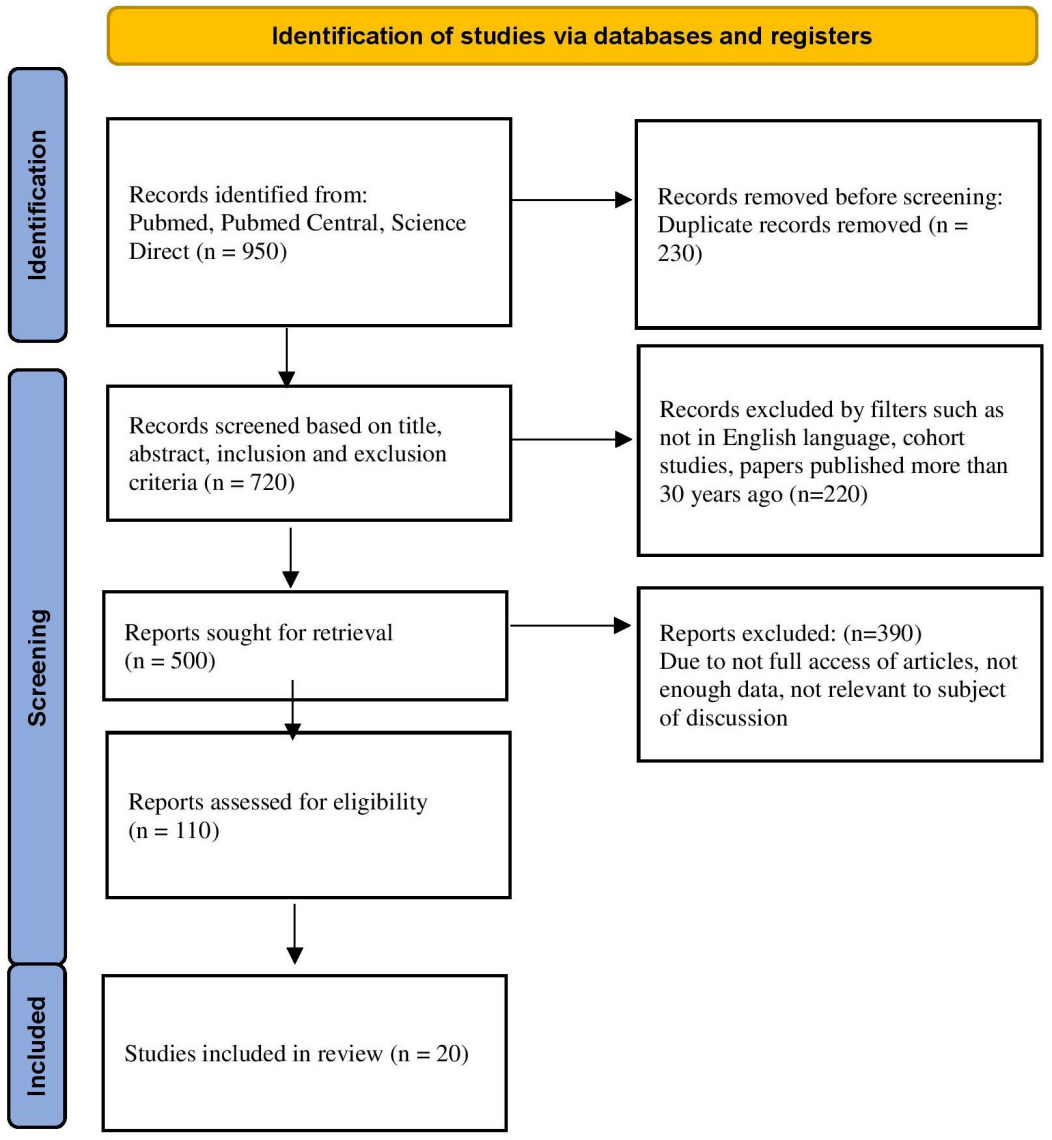
Prisma Flow Diagram.

Between January 1991 and December 2021, sources for this in-depth study were found using searches of PubMed, PubMed Central, and Science Direct, as well as references from pertinent papers for this systematic review. “Kawasaki,” “Strawberry Tongue,” “Rash,” “Inflammation,” and “Japan” were utilized as search phrases along with the conjunctions “OR” and “AND.” Articles in the English language, from the previous 30 years, and those in the pediatric and adult population were included after prescreening. Titles and abstracts were examined one by one to only include research that was pertinent to the subject. PRISMA guidelines were implemented for the same ( Figure 1). Inclusion and exclusion criteria are mentioned in Table 1.

**Table 1.** Inclusion and exclusion criteria.

Inclusion criteria	Exclusion criteria
Studies focussed on pediatric and adult individuals	Studies in geriatric population
Published Systematic Reviews, meta-analyses, case reports, literature reviews, randomized controlled trials, animal experiments, and prospective studies.	Cohort studies
Papers in the English Language	Papers not in the English language.
Papers selected from the previous 30 years	Papers published over 30 years ago.
Papers relevant to the subject of discussion.	Papers irrelevant to the subject of discussion.

## Discussion

### Epidemiology

Since 1970, countrywide surveys in Japan have been conducted to determine the frequency and incidence of KD. Seventy percent of all KD cases occur in children under three, according to the age distribution at the beginning of the disease, which peaks between 9 and 11 months old. According to the most recent study, more than 2,40,000 registered patients are there overall of this illness. In Japan, the number of youngsters with KD has been rising. ^3^ The surveys also revealed the following: (i) there have been three previous nationwide epidemics; (ii) there hasn't been a recent nationwide epidemic, but there is a small, localized epidemic that is spreading to the nearby region; (iii) the number of patients rises in the winter and falls in the summer; and (iv) Compared to non-siblings, siblings have a far higher chance of transmitting an infection. There are 220 instances per 100,000 children under the age of five in Japan, compared to 100 per 100000 on the peninsula. The prevalence is 10-20 times greater in Western countries. ^3^

Racial characteristics are more important than geographic ones in determining the status of KD. Hawaii's mean rate is 40, although racial disparities are evident; the rates for Japanese, Hawaiians, Chinese, Filipinos, and Caucasians are 360, 95, 77, 56, and seven respectively. ^3^ Furthermore, second-generation KD patients are significantly more numerous than expected, and the relative risk for siblings is ten times higher than expected. A solution to unlocking the riddle of KD may lie in the discovery of genetic elements linked to individual status or radically distinct occurrences among races. ^3^

**Figure 2.**
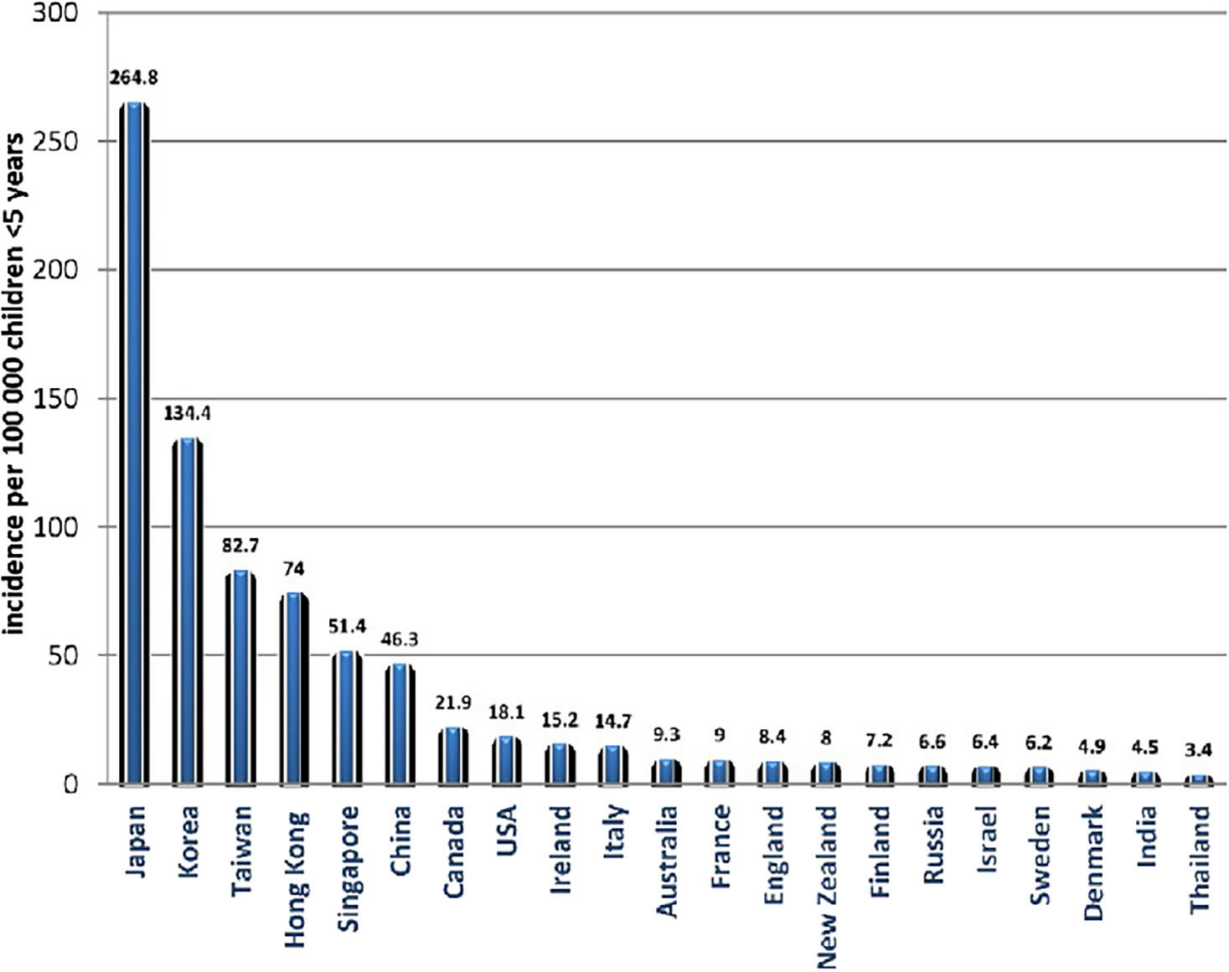
The global incidence of Kawasaki disease per 100,000 children under 5 years old (Figure from Elakabawi *et al.*, 2020). ^4^

According to one another study, the prevalence of KD in children under the age of five varies between 5 and 22 per 100,000 in North America, Australia, and Europe. Up until the last 10 years, when the rate has essentially stabilized, the incidence in these nations was rising, primarily because of increased ascertainment. In contrast, North-Eastern Asian nations, particularly Japan, Korea, and Taiwan, record an incidence that is almost ten times higher than that of Europe, Australia, and North America, and it has been increasing over the past 20 years. The prevalence appears to be significantly increasing and matches the epidemiological trends of the North-Eastern Asian nations in quickly developing economies like China and India, despite the epidemiological data being less precise ( Figure 2). ^4^

The prevalence of the second case illness of Kawasaki disease/mucocutaneous lymph node syndrome among 1788 siblings of children with the illness was calculated using information from questionnaires sent to members of the Japanese Association of Parents of Children with the Syndrome. When the first case in a family occurred at one-year intervals, the general second-case rate for siblings was 2.1%, compared to a corresponding overall incidence of roughly 0.19% in Japan's general population of children ages 0 to 4 during the epidemic year 1982. It was 8.4% for siblings under one year old and 9.3% for those between 35 and two years old. When the initial instances occurred, more than 54.1% of the second cases had already developed in ten days or fewer. ^5^

### Pathogenesis

In a study of 169 children in an urban centre county, environmental and alternative factors caused the KD. Higher rainfall and lower temperatures were associated with a higher incidence of KD, therefore it appeared that climatic conditions might also be important. The authors were able to control for several seasonality factors since the same connections were validated in the Japanese population using data from thirteen years' worth of countrywide surveys. KD has been proved to be associated with Tropospheric wind as well, with substantial changes in a pattern resembling peaks in KD incidence. A follow-up study suggested that some plant species or other environmental contaminants may be carried by easterly winds coming from northern China and cause KD. Four individuals were found to have evidence of viral infection likely related to KD after numerous considerations of typical background pathology as probable contamination with this sensitive approach. Bocavirus, rhinovirus, infantile paralysis virus, and contagion virus were the illnesses that were known. The number of factor variations linked to KD has significantly decreased as a result of genome-wide association studies.

In turn, these discoveries have opened up fresh perspectives on the pathophysiology of KD. Examples include one polymorphism within the FCGR2A factor and two susceptibleness loci that were known from one of the most significant social cohort studies. The FcRIIA receptor, a low-affinity Ig Fc receptor, is encoded by this factor. Immune cells, particularly phagocytes, express FcRIIA in a healthy manner. Following this, many FCGR2A polymorphisms have been linked to KD susceptibility in a variety of ethnic groups. Given the relevance of IV IG in the treatment of KD and the ongoing lack of a thorough understanding of the mechanism behind IV IG efficacy in KD, the connection of FCGR2A with risk for KD is particularly intriguing. Notably, IVIG resistance is correlated with high levels of FcRIIA messenger RNA expression. The second locus discovered in this study was ITPKC, a factor previously related to KD susceptibility in a disproportionately Japanese population. The ITPKC factor encodes B-1,4,5-triphosphate-3-kinase C, an association in nursing protein that reduces the calcium response in immune cells. Mixed inflammatory infiltrates comprising PMNLs, eosinophils, macrophages, and lymphocytes are detected affecting the tissue layer of the main coronary arteries in the early stages of the illness (about the first 10 days). The media is relatively unaffected. This severe inflammatory stage is characterized by perivasculitis, which also affects the blood vessels and microvessels. ^6^

### Immune response

Each innate and resolving immune cell gets activated and infiltrated into the arterial blood vessel wall as part of the disease's progressive response. A tube-shaped structure pathology has been categorized into three successive related with the diseased processes on the basis of examinations of post-mortem tissue from individuals with this disease. Within the first two weeks of the sickness, necrotizing redness appears and is caused by neutrophil infiltrations that gradually obliterate the media, tissue layer, and some deeper tissue layers of the arterial blood vessels. ^7^ Leucocyte infiltration, however, was rapidly followed by white blood cell infiltrates throughout a series of six specimens, and then mixed white blood cell and plasmacytic infiltrates became undeniable later, close to day nineteen of the illness. Along with induration of the arteries 39 plaque development, distinct nodular infiltrates have also been seen; however, they tend to develop at later time periods (>3 weeks). T cells, macrophages, B cells, and IgM+ plasma cells, which were more prevalent than IgA+ plasma cells, made up these infiltrates. ^8^ The number of IL-1-related genes and raised levels of IL-1 pathway proteins in plasma provide strong evidence that the lymphokine (IL-1) pathway is activated in KD. The IL-6/T helper TH-17 axis 21 and the IL-12/interferon-gamma axis are two major protein clusters identified in KD. Naive T cells are polarised toward a Th-17 composition by IL-6 and remodelling protein beta (TGF), 21 which results in these cells penetrating the vessel wall and altering the protein profile. ^9^ A CD4:CD8 ratio of 2.3-2.7 in the peripheral blood in acute KD indicates a substantial reversal of the CD4:CD8 ratio in the disease's principal target tissue. ^10^

### Symptoms

Patients will have a fever of at least 100.4°F for at least five days. The duration of a fever without treatment might reach eleven days. There are at least four of the following symptoms in addition to the fever:

i) A rash covering the body's extremities and the trunk.
ii) The palms and soles of the feet become red and edematous. Within the second and third weeks, the skin on the fingers and toes begins to peel.
iii) Red eyes that might be light-sensitive.
iv) Mouth, lip, and throat irritation and inflammation. A “strawberry” tongue is recognizable because it has larger style buds and is joltingly red.

Abdominal discomfort is a potential symptom for patients. Only a tiny percentage of individuals experience transient inflammatory diseases that cause knee and hip pain and swelling. If a newborn has fever and inflammation but none of the other signs are present, incomplete illness should be taken into consideration. ^11^ Additionally, there may be bilateral non-exudative conjunctivitis. ^12^

### Neurological involvement in Kawasaki disease

Neurologic involvement in KD is reported in 1.1% to 3.7% of KD cases. ^13^ It ranges from common symptoms like irritability and lethargy to multi-system involvement such as seizures, myositis, sterile infectious diseases, facial palsy, hemiplegia, and coma. The role of single-photon emission computed imaging (SPECT) is rising in the analysis of cerebral hypoperfusion in KD secondary to cerebral rubor. ^14^ Facial nerve paralysis is predominant in females and most facial paralysis is unilateral, and mostly on the left side. ^15^^–^^16^

Cerebral infarction may be a rare complication of KD and happens within the acute or subacute stage, A case report in a 5-month-old male patient with KD showed that even after receiving timely intravenous immunoglobulin therapy, he developed coronary artery aneurysms (CAA) and coronary artery thrombosis (CAT) one month later. Medical aid and thrombolytic agents were suggested, however, the child’s parents refused. Fifteen months after KD’s onset, an associated attack of syncope left him with left hemiplegia. CT scans revealed cerebral infarction of the right basal ganglion without hemorrhage. ^17^ Cerebral infarction in KD is usually attributed to (1) inflammatory pathology or occlusion of cervical or intracranial large-sized vessels, or (2) occlusion from hypokinetic cardiac muscle. ^18^

The neurological manifestations in KD can present as headache, convulsions, somnolence, extreme irritability, signs of membrane irritation, bulging fontanelles, and facial palsy. ^19^ Histopathological studies from postmortem examinations of KD patients have shown neuritis and ganglionitis of bones and peripheral nerves, aseptic meningitis, and meningitis. It's been believed that vasculitis of the vessels supplying the nerves could account for the impact on peripheral nerves. A report of the coincident development of migraine and Raynaud’s development twelve years after the development of KD suggested that KD might result in endothelial tissue pathology dysfunction as a late complication. ^20^

Acute encephalopathy, subdural effusion, and dyssynergia have also been reported. ^21^

### Complications

i) Myocarditis, endocarditis, pericarditis.
ii) Congestive heart failure
iii) The CNS, RS, musculoskeletal system, and other bodily systems may all be affected by Kawasaki illness.
iv) Coronary artery aneurysms, calcifications, strictures, and dysfunction brought on by scarring of the leaflets or progressively dilated artery roots. ^22^
v) Coronary artery thrombosis-induced occlusion. ^23^

### Diagnosis

A medical expert should evaluate the child and look for symptoms and indications that are typical with KD. Although these tests aren't specific for KD, blood testing and other serological tests can be performed to rule out alternative disorders and the presence of inflammation. The coronary arteries and the way the valves are working may both be clearly seen in an Ultrasonography scan. This could help in identifying the disease early to avoid any sequelae. ^24^

### Biomarkers

Early diagnosing is vital for the prompt establishment of intravenous gamma globulin medical treatment in KD. ^25^ The ordinarily used inflammatory markers, (ESR), (CRP), and total leukocyte counts (TLC) lack specificity for KD. ^26^ CASP5 (Caspase 5) and CR1(Complement C3b/C4b Receptor 1) genes were shown to probably discriminate KD from alternative similar diseases and additionally from healthy folks. ^27^ Solely four laboratory markers at presentation correlate with CAA development in KD: lower platelets, high neutrophils, high lymphocytes, and high C-reactive protein. ^28^ So as to understand the diagnostic serum biomarkers for KD, a previous study explored serum KD-related proteins, that were differentially expressed throughout the acute and recovery phases of two patients by mass chemical analysis (MS). The degree of 3 of those proteins, particularly lipopolysaccharide-binding macromolecule (LBP), leucine-rich alpha-2-glycoprotein (LRG1), and angiotensinogen (AGT), were higher in acute section patients. Also, the amount of retinol-binding macromolecule four (RBP4) was ablated. ^29^ Lipoprotein receptor eleven (LR11) may be a member of the beta-lipoprotein receptor family, which is expressed markedly in membrane vascular SMCs and secreted in a very soluble type (sLR11). sLR11 has been recently known as a possible vascular lesion biomarker and is reportedly elevated in patients with arterial blood vessel chronic lesions, however, there's no description of sLR11 in acute KD. sLR11 also can mirror the state of acute KD and could be a biomarker for patient response to IVIG medical aid. ^30^ Differentially expressed proteins (DEPs) in KD as compared to traditional controls, additionally created the functional annotation and protein interaction (PPI) networks in KD and respiratory disease. Forty-three and sixty-two DEPs were known in KD and respiratory disease, respectively. Serinehydroxymethyl transferase may be an important hub macromolecule of the KD-specific PPI network. ^31^ Serum ferritin is understood to be a helpful biomarker for the prediction of IVIG-nonresponsive KD, coronary artery abnormalities (CAAs), and macrophage activation syndrome (MAS) tendencies. ^32^ In a study, carboxyterminal propeptide of procollagen type I (PIPC), soluble suppressor of tumorigenicity II, galectin-3 (Gal-3), and growth-differentiation factor-15 were associated with the late convalescent blood cultures from a group of sixteen KD patients, who had an ejection fraction of less than 55% on their initial echocardiogram test. Results were analyzed and compared with blood samples from similar sex and age KD individuals having initial ejection fraction of more than 60%. According to the univariate analysis of the groups, the median Gal-3 and levels of PIPC within the low ejection fraction cluster were considerably much higher than those within the traditional ejection fraction cluster. Convalescent KD individuals with a history of low ejection fraction throughout the acute sickness had considerably increased levels of Gal-3 and PIPC as compared to that in the KD patients in the second group. These findings indicate the possible development of fibrosis as a late abnormalcy of severe carditis which might develop as sequelae to KD. ^33^ Also, a set of five genes are candidates as biomarkers for KD diagnosing, namely, the HLA-DQB1, HLA-DRA, ZBTB48, TNFRSF13C, and CASD1. ^34^

### Treatment

The use of high-dose IV IG is recommended by the most recent management recommendations for mucocutaneous lymph node syndrome. If administered for the first ten days of the illness, IV IG lowers the risk of coronary rubor and aneurysm development. Although the exact methods by which the IV IG therapy lowers inflammatory responses are yet understood, it is believed to have a broad range of activity that targets several immune response pathways. It has been demonstrated that IV IG inhibits macrophages' ability to produce IL-1 in vitro and promotes the assembly of IL-1Ra in mucocutaneous lymph node syndrome. By saturating Fc receptors, ^35^ it also lessens the synthesis of chemokines and pro-inflammatory cytokines as well as the activation of neutrophils, monocytes, macrophages, and T cells. ^36^

In research, clarithromycin and endogenous antibody were used, and the results revealed that clarithromycin lowers the KD relapse rate. Through its anti-biofilm function, clarithromycin appears to be useful in preventing KD recurrence by preventing the release of bioactive chemicals. Clarithromycin may thus be beneficial in treating people with the acute form of KD as well as their siblings to stop the disease from spreading to them. ^37^ There were fewer refractory KD patients in the combination therapy cluster than in the IV IG alone cluster in a trial of two groups, one receiving an anti-TNF drug along with IV IG, and the other receiving IV IG alone. Patients with KD who received combination therapy had better results, shorter hospital stays and fever durations, and less arterial dilatation. ^38^ Children with KD can get safe and effective antithrombotic treatment using clopidogrel in combination with a low-dose anodyne. ^39^ The combination of the aforementioned medication with a lipid-lowering drug with immunomodulatory effects has the potential to be beneficial for children with acute mucocutaneous lymph node syndrome and early symptoms of coronary artery damage. ^40^

### Prognosis

When a child is detected with Kawasaki illness, the degree and severity of coronary arteries damage at diagnosis and during evaluation are the main factors determining prognosis. The most common cause of death is coronary artery disease leading to myocardial infarction brought on by the blockage of the vessel, and the actual case-fatality rate in Japan and the US is less than 0.2%. A thorough risk classification methodology is provided by the AHA standards when it comes to the assessment, cure, and management of KD, ^41^ which can be used as follow-up counseling. Five risk groups are used in the classification scheme, which uses both absolute luminal measurements as well as Z scores. When the coronary arteries are not involved, the danger level is 1 (Z score 2). Both during the acute sickness and 6–8 weeks after the disease started, these individuals are examined with echocardiograms. These patients resemble people without a KD diagnosis in terms of risk assessment. ^42^ As long as, in these individuals, the risk classification does not alter negatively, ASA medication can be stopped in this group. Risk category 5 having large or massive aneurysms is the category with the greatest risk. These patients need considerably more frequent cardiac monitoring and may potentially need to take anticoagulants if their aneurysms continue to develop as described above. ^43^

## Conclusion

The diagnosis, immediate care, and long-term therapy of KD are discussed in this article along with updated suggestions. The avoidance of significant coronary artery anomalies is the ultimate objective. The difficulties of an isolated incident of KD throughout childhood are thought to be the cause of the unexpected death or CAD that younger adult patients are continuing to present with. Even though the finest available information and professional opinion were used to construct this review, significant evidence gaps were found. A specific diagnostic test continues to be difficult, and acute management is rather empirical until the etiology and pathophysiology of this condition are identified properly and early. Furthermore, there is no way for us to prohibit KD. A tiny fraction of individuals may develop facial paralysis, cerebral infarction, and hemiplegia. Some may have coronary artery aneurysms when they are first diagnosed or develop them despite the most advanced empiric treatment now available, to which some individuals do not react.

## Data Availability

No data are associated with this article.
