# Supplementary material for: Neurological involvement, immune response, and biomarkers in Kawasaki disease along with its pathogenesis, therapeutic and diagnostic updates
Source: F1000Res. 2023 Apr 3;12:235. Originally published 2023 Mar 2. [Version 2] doi: 10.12688/f1000research.130169.2 (PMC10102713; doi:10.12688/f1000research.130169.2)
Supplement: Peer review history

# Peer review: Reviewer response for version 2

**DOI:** 10.5256/f1000research.145895.r168598

**Published:** 2023-04-13

**Authors:** Arpit Mago

**Affiliations:** 1 Department of Clinical Medicine, Jawaharlal Nehru Medical College, Belgaum, Karnataka, India

I have gone through the changes made by the authors and I am satisfied with them. My decision is to approve the article with no further changes required.

Is the review written in accessible language?

Yes

Are all factual statements correct and adequately supported by citations?

Yes

Are the conclusions drawn appropriate in the context of the current research literature?

Partly

Is the topic of the review discussed comprehensively in the context of the current literature?

Yes

Reviewer Expertise:

NA

I confirm that I have read this submission and believe that I have an appropriate level of expertise to confirm that it is of an acceptable scientific standard.

# Peer review: Reviewer response for version 2

**DOI:** 10.5256/f1000research.145895.r168599

**Published:** 2023-04-04

**Authors:** Alhad Mulkalwar

**Affiliations:** 1 Department of Medicine, Seth Gordhandas Sunderdas Medical College, King Edward Memorial Hospital, Mumbai, Maharashtra, India

The newer version is quite enhanced in presentation and ease of readability from the previous version. The neurological involvement shift with symptoms and biomarkers shift below diagnosis makes it easier to comprehend the article. The addition of global incidence figure and text adds important value to the manuscript.

Is the review written in accessible language?

Yes

Are all factual statements correct and adequately supported by citations?

Yes

Are the conclusions drawn appropriate in the context of the current research literature?

Yes

Is the topic of the review discussed comprehensively in the context of the current literature?

Yes

Reviewer Expertise:

Internal Medicine, Research

I confirm that I have read this submission and believe that I have an appropriate level of expertise to confirm that it is of an acceptable scientific standard.

# Peer review: Reviewer response for version 1

**DOI:** 10.5256/f1000research.142903.r165334

**Published:** 2023-03-20

**Authors:** Arpit Mago

**Affiliations:** 1 Department of Clinical Medicine, Jawaharlal Nehru Medical College, Belgaum, Karnataka, India

I would first like to congratulate the author team for their efforts in bringing a good research paper but I feel certain issues need to be addressed:

1. In the methods, type of research design should be clearly described.
2. There is a mismatch in the databases in the PRISMA Flow diagram and the ones mentioned in the text, it needs to be correct. Also the total reports sought for retrieval mentioned are 500 but in the second step only 120 are mentioned to be removed out of 720, so the final number should 600. There should be a clear mention of where the other 100 articles were excluded.
3. There is no mention if case reports, letters and editorials which often talk about new advances in case of different diseases were included or not 
4. In table 1 it's mentioned that animal experiments are also included but in the text it's mentioned that only human studies were included. Please explain this statement. Besides last one, inclusion and exclusion is quite irrelevant and should be removed.
5. The discussion is heavily focussed on Japan, it should be more broad and compare the incidence of Kawasaki disease in different continents or at least in most countries that disease is prevalent.
6. Discussion is very unstructured. Biomarkers should be talked about under the diagnosis section as it's a component of it and should be clubbed with the treatment under management section.
  Neurological involvement is also just a separate heading breaking the flow of ideas, it should be properly included under the symptomatology section.
  Besides the pathology section should be more elaborate with mention of the inconsistencies reported in the pathology in other published articles.

Is the review written in accessible language?

Yes

Are all factual statements correct and adequately supported by citations?

Yes

Are the conclusions drawn appropriate in the context of the current research literature?

Partly

Is the topic of the review discussed comprehensively in the context of the current literature?

Yes

Reviewer Expertise:

immunogenetics and neurological manifestations

I confirm that I have read this submission and believe that I have an appropriate level of expertise to confirm that it is of an acceptable scientific standard, however I have significant reservations, as outlined above.

# Response

**Published:** 2023-03-20

**Authors:** Omniat Amir

**Affiliations:** Manhal Academy, Sudan

Hi, Thank you for your detailed and positive review.

 The methods section and Prisma diagram have been updated to include the details more clearly. Although previously too, the incidence of KD in some countries were talked about, now one more paragraph and a figure have been added comprehensively discussing the incidence of KD in various other countries. The biomarker subsection is shifted below the diagnosis subsection, and the Neurological involvement subsection is shifted below the symptoms subsection to facilitate easy understanding in the new version.

# Peer review: Reviewer response for version 1

**DOI:** 10.5256/f1000research.142903.r165333

**Published:** 2023-03-07

**Authors:** Alhad Mulkalwar

**Affiliations:** 1 Department of Medicine, Seth Gordhandas Sunderdas Medical College, King Edward Memorial Hospital, Mumbai, Maharashtra, India

The review discusses Kawasaki disease in good detail. The authors have included various subsections such as pathogenesis, neurological signs, immune response, diagnosis, biomarkers, complications, treatment, and prognosis. The article is well-summarised and scientifically accurate. For instance, it mentions the diagnostic criteria such as that patients should have a fever for at least five days of more than 100.4 F. It is usually accompanied by a rash, edematous palms, and soles, reddening of eyes, and strawberry tongue. The cerebral symptoms include infarction, stroke, paralysis, and facial palsy. Some places such as diagnosis and diagnostic criteria could have been described a bit more and be clearer. But, overall the article is well written and the subtopics are properly summarised as mentioned.

Is the review written in accessible language?

Yes

Are all factual statements correct and adequately supported by citations?

Yes

Are the conclusions drawn appropriate in the context of the current research literature?

Yes

Is the topic of the review discussed comprehensively in the context of the current literature?

Yes

Reviewer Expertise:

Internal Medicine, Research

I confirm that I have read this submission and believe that I have an appropriate level of expertise to confirm that it is of an acceptable scientific standard.
